# Supplementary material for: Comprehensive Annotation and Functional Exploration of MicroRNAs in Lettuce
Source: Front Plant Sci. 2021 Dec 24;12:781836. doi: 10.3389/fpls.2021.781836 (PMC8739914; doi:10.3389/fpls.2021.781836)
Supplement: Supplementary file 2 [file Data_Sheet_8.ZIP › GSM2230749.plot/Lsa-miR166i_Lsat_1_v5_gn_1_14320.1_473_TPlot.pdf]

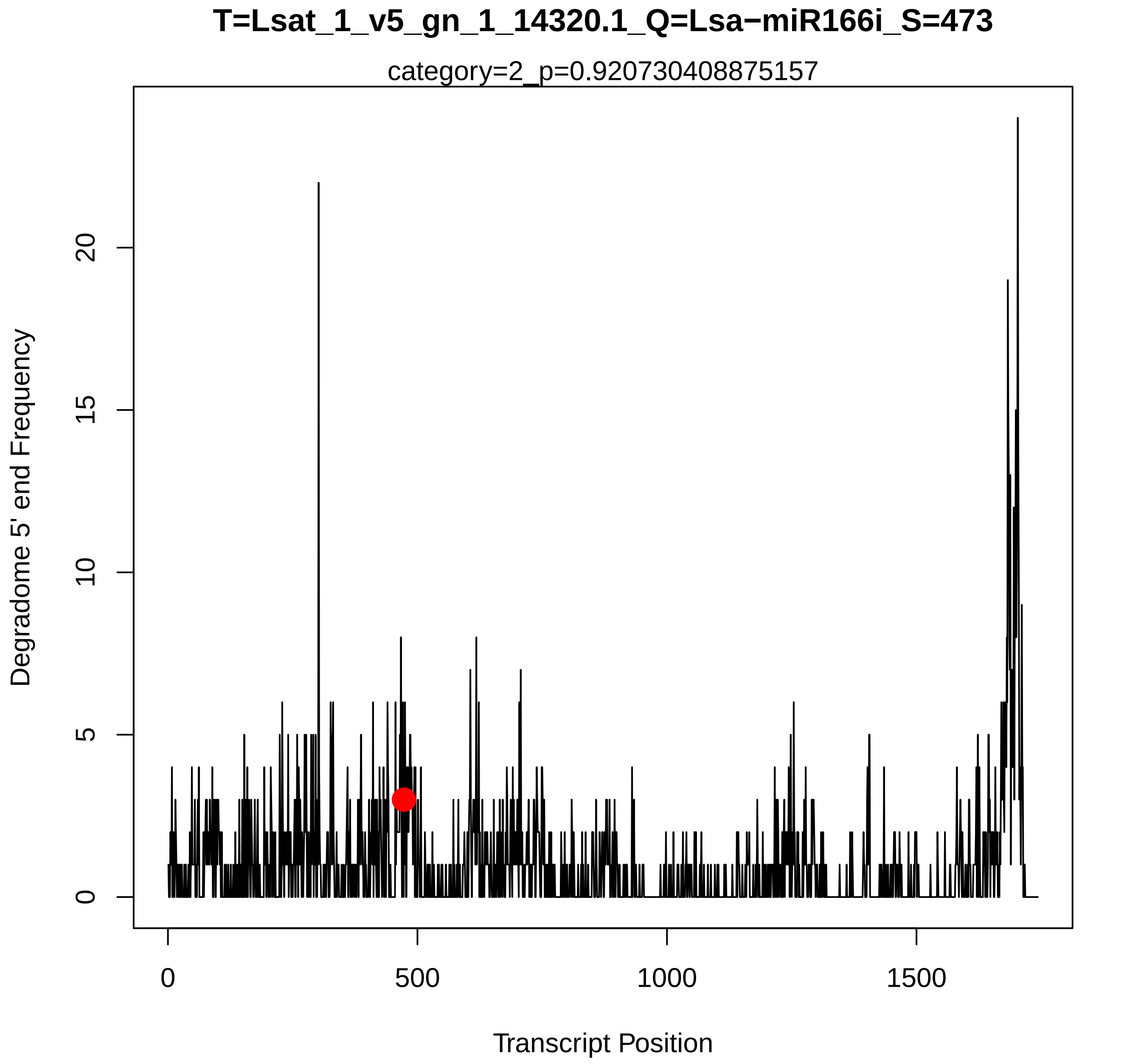
T=Lsat_1_v5_gn_1_14320.1_Q=Lsa−miR166i_S=473
category=2_p=0.920730408875157
Degradome 5' end Frequency
0
5
10
15
20
0
500
1000
1500
Transcript Position
